# Pluripotent Stem Cells in Clinical Setting—New Developments and Overview of Current Status

**DOI:** 10.1093/stmcls/sxac040

**Published:** 2022-06-07

**Authors:** Dusko Ilic, Caroline Ogilvie

**Affiliations:** Division of Women and Children’s Health, Faculty of Life Sciences and Medicine, King’s College London, London, UK; Assisted Conception Unit, Guy’s Hospital, London, UK; Genetics Laboratories, Guy’s Hospital, London, UK

**Keywords:** clinical trials, embryonic stem cells, induced pluripotent stem cells, pluripotent stem cells

## Abstract

The number of clinical trials using human pluripotent stem cells (hPSC)—both embryonic and induced pluripotent stem cells (hESC/iPSC)—has expanded in the last several years beyond expectations. By the end of 2021, a total of 90 trials had been registered in 13 countries with more than 3000 participants. However, only US, Japan, China, and the UK are conducting both hESC- and hiPSC-based trials. Together US, Japan, and China have registered 78% (70 out of 90) of all trials worldwide. More than half of all trials (51%) are focused on the treatment of degenerative eye diseases and malignancies, enrolling nearly 2/3 of all participants in hPSC-based trials. Although no serious adverse events resulting in death or morbidity due to hPSC-based cellular therapy received have been reported, information about safety and clinical efficacy are still very limited. With the availability of novel technologies for precise genome editing, a new trend in the development of hPSC-based cellular therapies seems to be emerging. Engineering universal donor hPSC lines has become a holy grail in the field. Indeed, because of its effectiveness and simplicity nanomedicine and in vivo delivery of gene therapy could become more advantageous than cellular therapies for the treatment of multiple diseases. In the future, for the best outcome, hPSC-based cellular therapy might be combined with other technological advancements, such as biomimetic epidural electrical stimulation that can restore trunk and leg motor functions after complete spinal injury.

## Graphical Abstract

**Figure:**
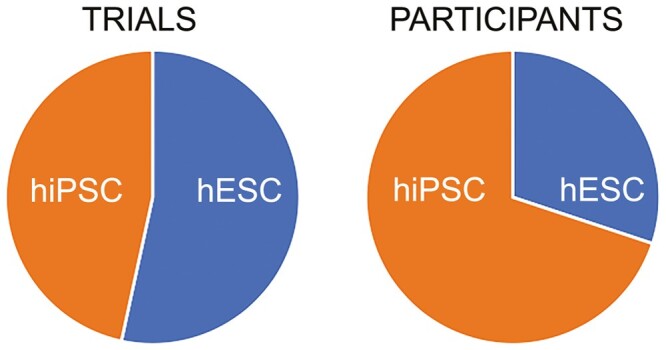

**Significance Statement**

The increase in the number of hPSC-based clinical trials, from 12 in 2015 to 90 in 2021, indicates that the field has matured enough to be taken seriously by Big Pharma and investors. Indeed, Fate Therapeutics is involved in 13, Astellas in 8, and ViaCyte in 5 clinical trials with 1587, 128, and 367 participants, respectively. The affordability of hPSC-based cellular therapies is likely to increase due to the development of universal donor iPSC lines for off-shelf treatment. The efficacy of hPSC-based cellular therapy might be improved in combination with other technological advancements.

In the last several years, the number of clinical trials with human pluripotent stem cells (hPSC)-based therapies is rapidly increasing, from 12 in 2015^1^ to 54 in 2019,^2^ and 90 in 2021 (Table 1, Fig. 1A). Although there are more human embryonic stem cells (hESC)-based trials, the number of participants enrolled in human induced pluripotent stem cells (hiPSC)-based trials is nearly 2-fold higher (1942 vs 979) (Fig. 1B, 1C). In a year or two, hiPSC-based trials will probably take over.^1^ Thirteen countries reportedly run hPSC-based clinical trials, although 78% of these trials (70 out of 90) are conducted in just three of these countries: US (35), China (17), and Japan (18). US, China, Japan, and the UK are the only countries conducting both hESC- and hiPSC-based trials.

**Table 1.**
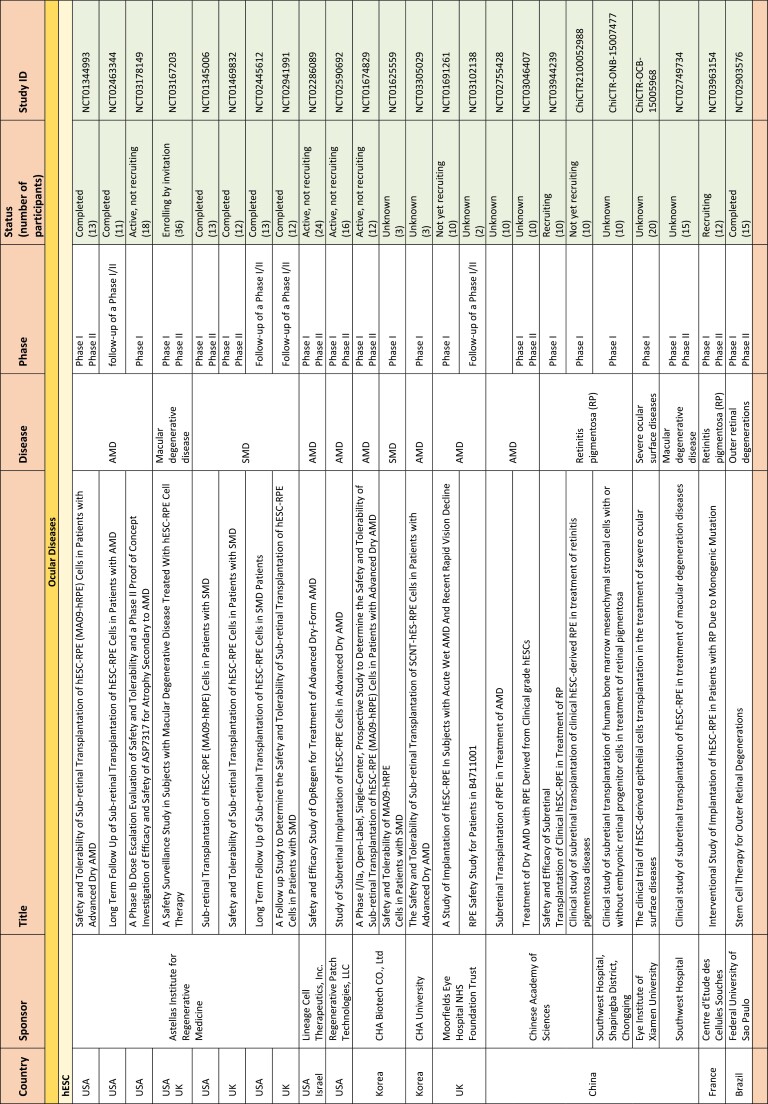

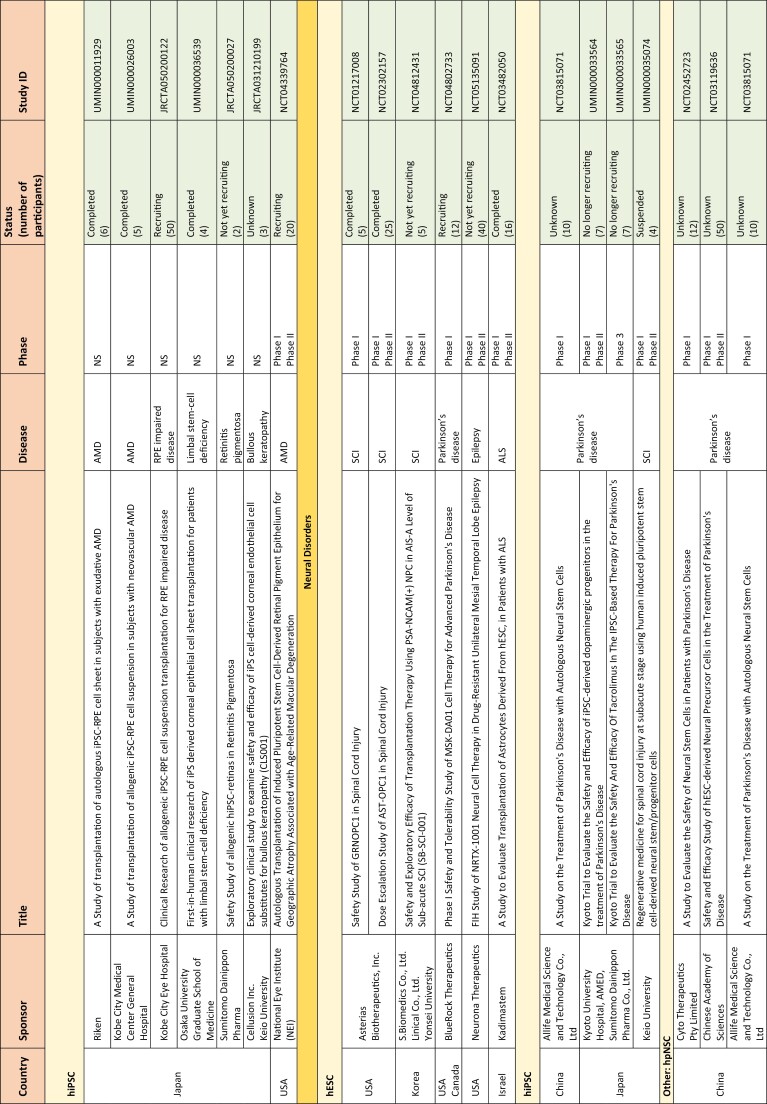

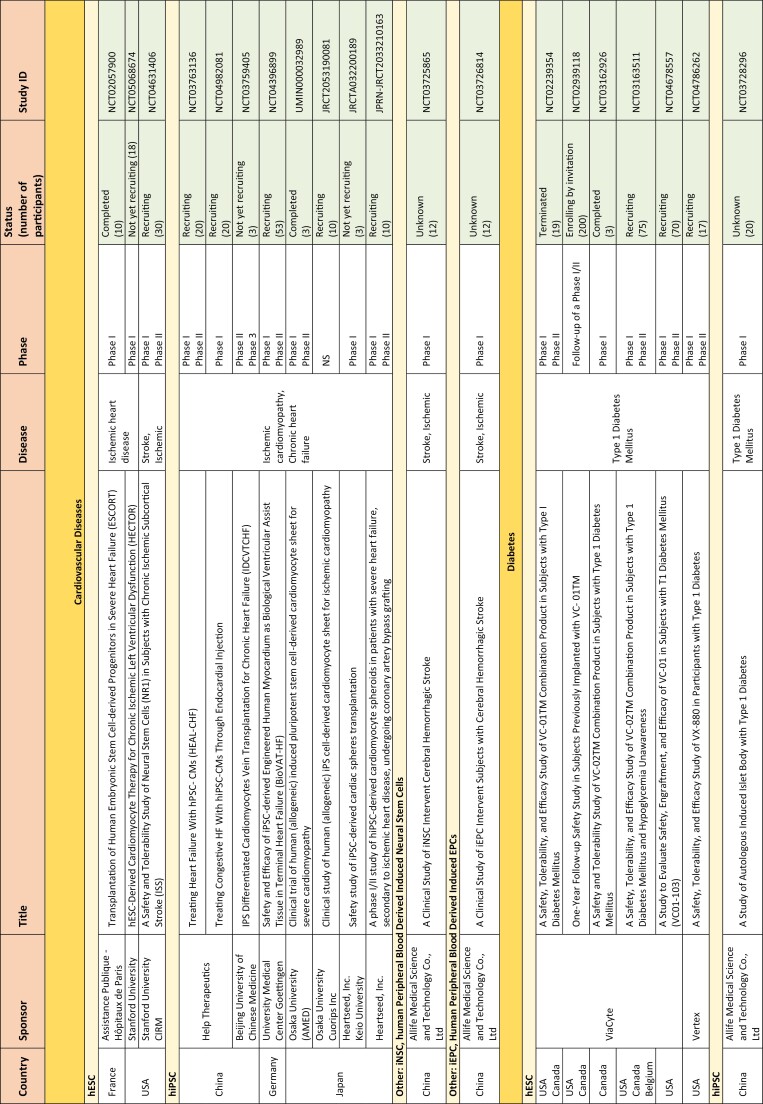

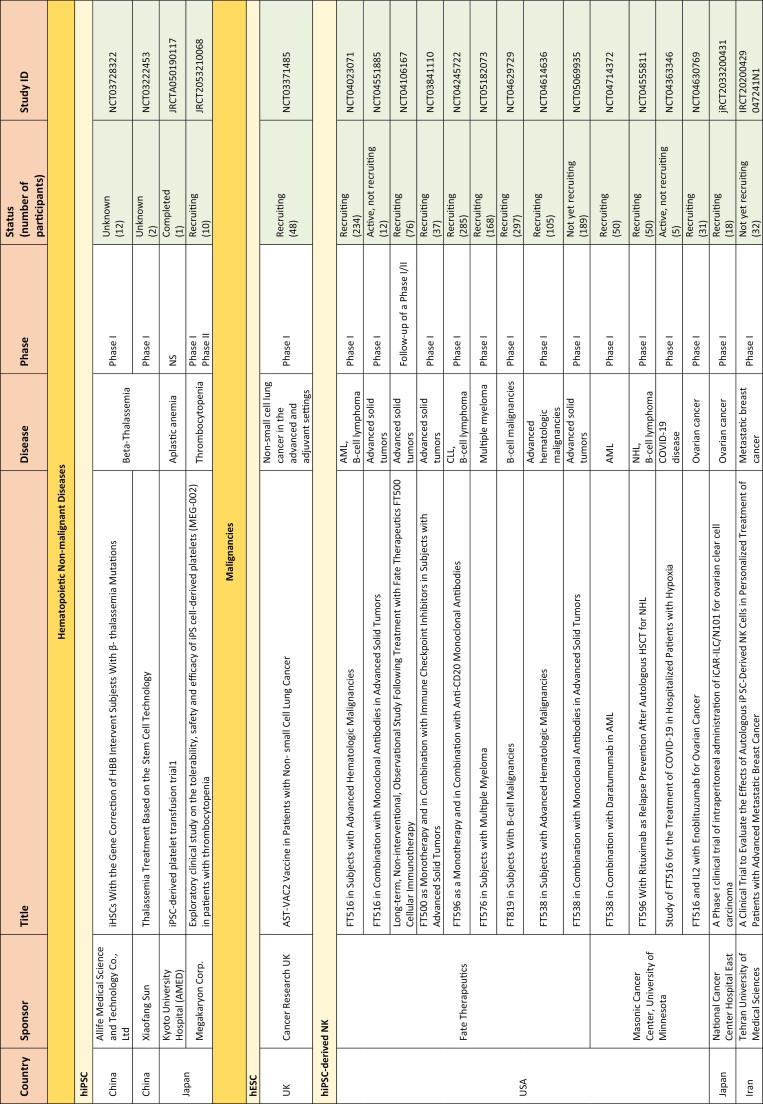

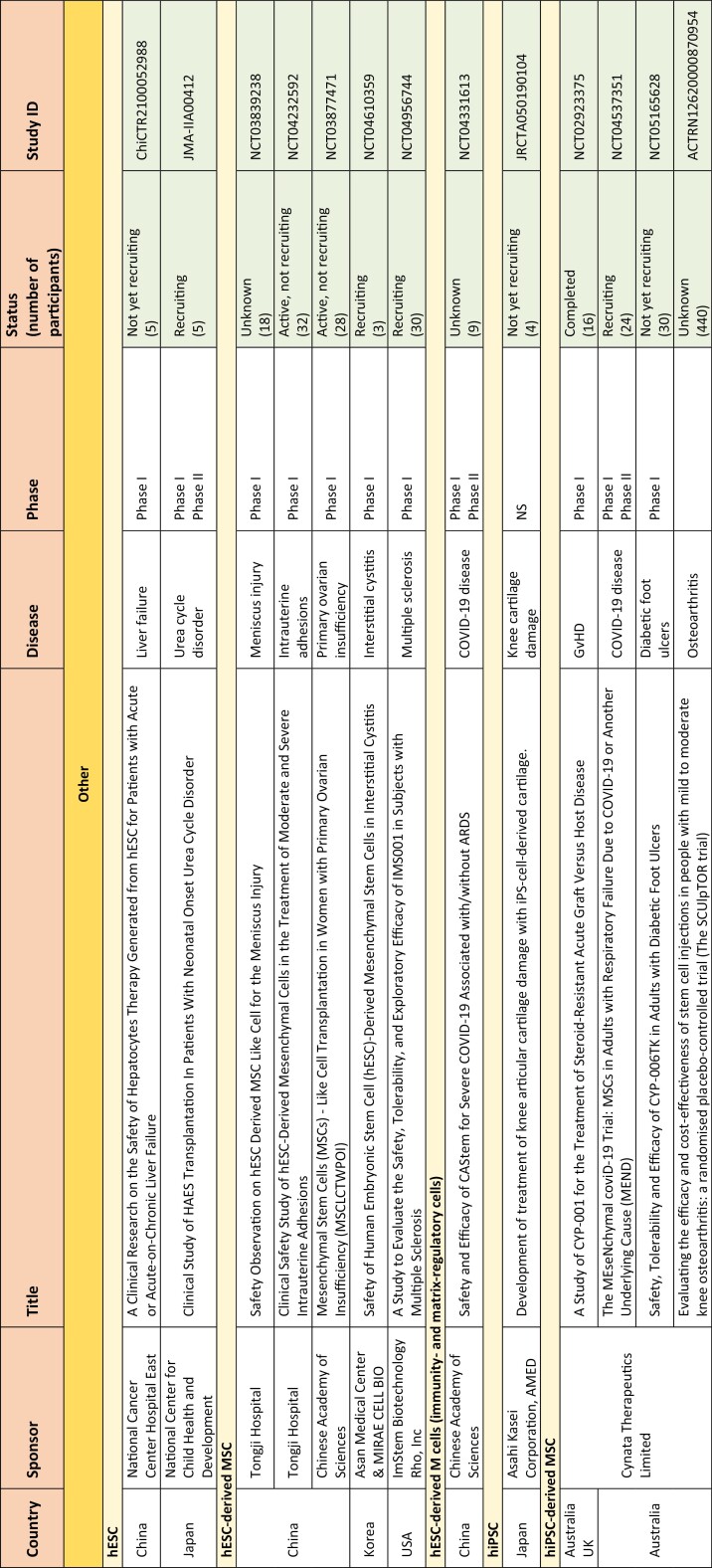
hPSC-based clinical trials by December 31, 2021.

Abbreviations: ALS, amyotrophic lateral sclerosis; AMD, age-related macular degeneration; AML, acute myeloid leukemia; CLL, chronic lymphocytic leukemia; GvHD, graft versus host disease; hESC, human embryonic stem cells; hiPSC, human induced pluripotent stem cells; hpNSC, homogeneous population of multipotent neural stem cells; iNSC, induced neural stem cells; MSC, mesenchymal stem cells; NHL, non-Hodgkin lymphoma; NS, non-significant; RPE, retinal pigment epithelium; SCI, spinal cord injury; SMD, Stargardt’s macular dystrophy.

**Figure 1.**
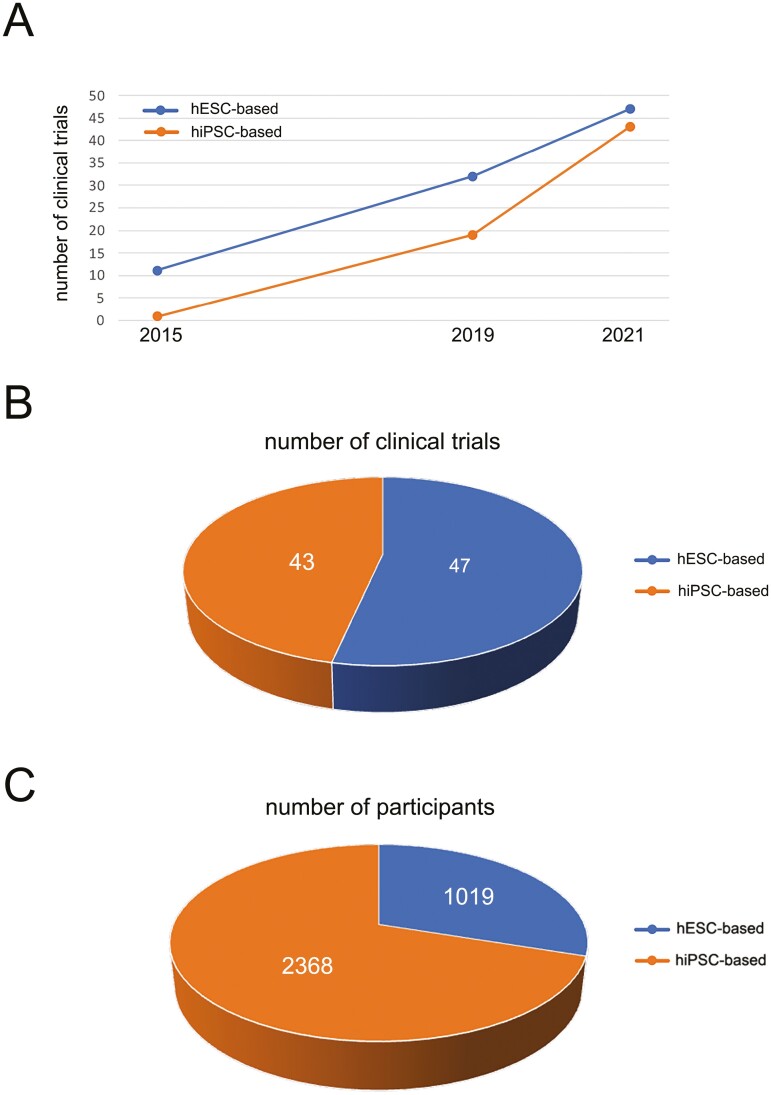
Clinical trials with hPSC-based therapies. **(A):** Number of clinical trials is rapidly increasing from 12 in 2015 to 54 in 2019, and 90 in 2021. **(B):** hESC-based clinical trials are still prevailing over the iPSC-based (47 vs 43). **(C):** Number of participants is higher in iPSC- than hESC-based clinical trials (2368 vs 1019).

The trials are focused mainly on four areas: degenerative diseases of the eye (30), malignancies (16), neural degenerative disorders (11), and cardiovascular diseases (10). Clinical trials for treatments of degenerative diseases of the eye, neural degenerative disorders, and type 1 diabetes are predominantly hESC-based, whereas cardiovascular diseases and malignancies are hiPSC-based (Fig. 2A). The highest number of participants (1637) were enrolled in hPSC-based treatment of malignancies, followed by degenerative diseases of the eye (407) and type 1 diabetes (405) (Fig. 2B).

**Figure 2.**
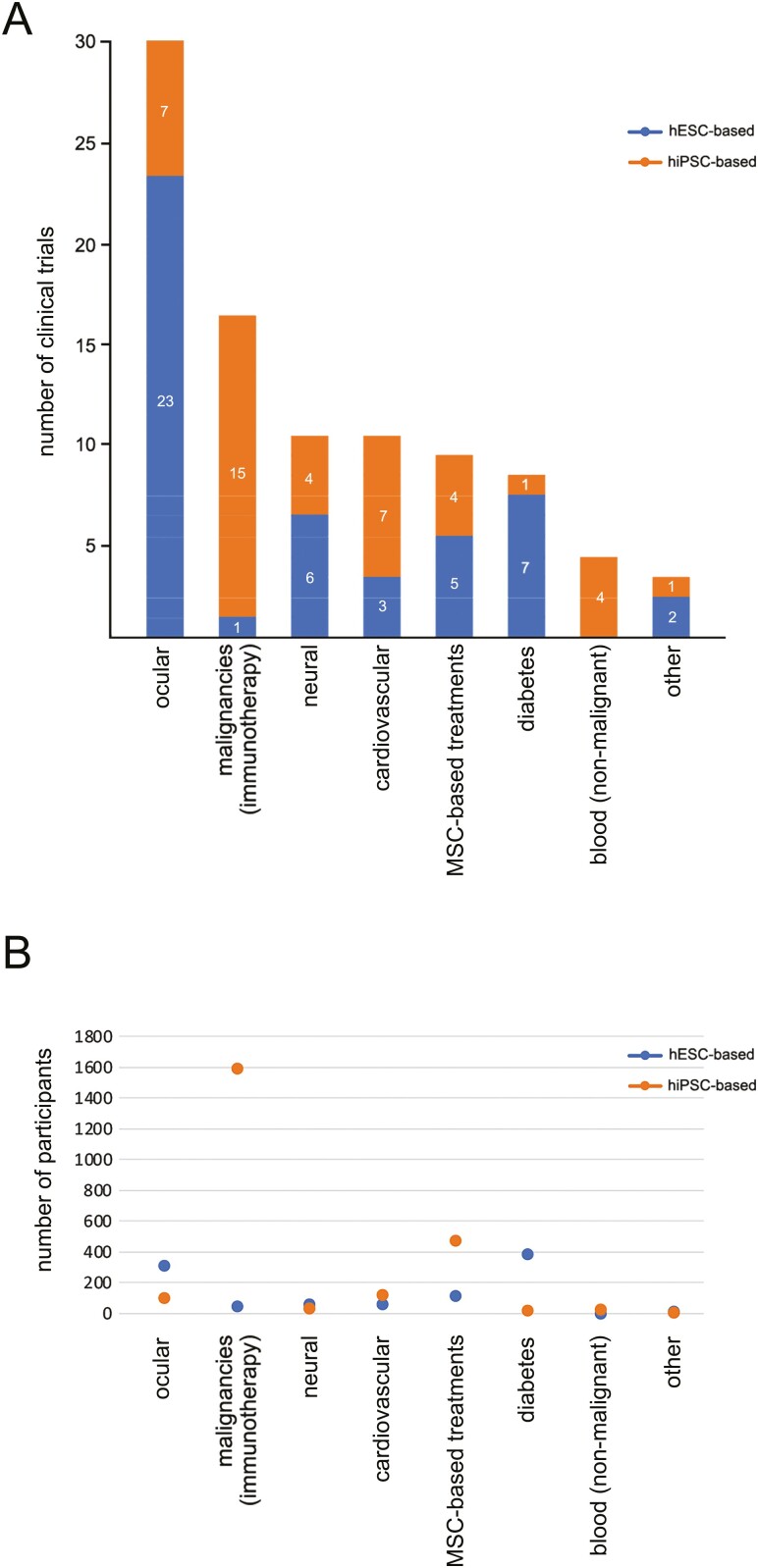
Distribution of hPSC-based clinical trials per condition treated. **(A):** Number of trials per condition treated. **(B):** Number of participants enrolled in hPSC-based clinical trials per condition treated.

The information summarized here may not be complete and/or fully accurate. We have collated data from the following databases: US Clinical Trials (http://clinicaltrials.gov), EU Clinical Trials Register (www.clinicaltrialsregister.eu), Human Pluripotent Stem Cell Registry (hPSC^reg^; https://hpscreg.eu/browse/trials), Australian Clinical Trials (www.australianclinicaltrials.gov.au), Chinese Clinical Trial Registry (www.chictr.org.cn/enIndex.aspx), International Clinical Trials Registry Platform (ICTRP; www.who.int/clinical-trials-registry-platform), and the Japan Primary Registries Network Search Portal (https://rctportal.niph.go.jp/en/link), which covers the registries of four institutions: Ministry of Health, Labour and Welfare (JRCT), the University Hospital Medical Information Network Center (UMIN-CTR), the Japan Pharmaceutical Information Center (JAPIC), and the Japan Medical Association Center for Clinical Trials (JMACCT). Information were not always matched between databases. For example, cell therapy for advanced Parkinson’s disease sponsored by *BlueRock Therapeutics* has a target of 12 participants on the US Clinical Trials site (NCT04802733), whereas on hPSC^reg^ the target is 10; two studies evaluating treatment of Parkinson’s disease sponsored by Kyoto University were still active according to JMACCT, and not recruiting according to UMIN-CTR. Despite discrepancies, the presented overview largely reflects the current picture of hPSC-based clinical trials worldwide. We have also listed in the table three trials from China with insufficient information for full classification: induced neural stem cells (iNS) and induced endothelial progenitor cells (iEPC), both derived from the peripheral blood, and M cells or immunity and matrix-regulatory cells derived from hESC. Some of the information discussed has not been peer reviewed (eg, press releases or conference abstracts) and could not be independently verified.

## Spinal Cord Injury—New Beginnings

In October 2010, the first patient was treated with hESC-based therapy at Shepherd Center, a 132-bed spinal cord and brain injury rehabilitation hospital and clinical research center in Atlanta, Georgia.^3^ This was the first hPSC-based clinical trial worldwide. The trial was run by the California-based company *Geron*, and in phase I of the trial, 2 million oligodendrocyte progenitors were transplanted into the site of subacute spinal cord injury (SCI).^4^ Although the initial data were encouraging and safety was demonstrated, the trial was abandoned after a year; the therapy did not show any signs of efficacy.^5^ Another company, *Asterias Therapeutics*, acquired the technology and continued where *Geron* had stopped; in 2019, the Company reported the results from a trial using 5-10× higher doses of 10-20 million cells.^6^ The higher doses were also safe, and no adverse events associated with the therapy were reported. The results were quite different from *Geron*’s trial—95% of these patients demonstrated improved sensory and motor function, indicating that a dose of 2 million cells was too low, and that at least 5× more cells should be transplanted to see any effect.

In 2021, a Japanese team published a design of a clinical trial treating patients with SCI with hiPSC-derived neural stem/progenitor cells (NS/PCs).^7^ Disappointingly for the patients, the dose in phase I of the clinical trial was again 2 million cells. Even though plans to run dose-escalation trial are in place, the question remains is this subtherapeutic starting dose necessary, especially after the recently reported successful outcome of SCI treatment using a completely different approach.^8^ This approach using only epidural electrical stimulation (EES) targeting the dorsal roots of lumbosacral segments, delivered with a multielectrode paddle, restored walking in patients with SCI with complete sensorimotor paralysis. Activity-specific stimulation programs enabled the three patients on which the device has been tested to stand, walk, cycle, swim, and control trunk movements in a single day.

Although the patients could move independently, the movements were not natural; they were enabled via biomimetic stimulation programs. During a 5-month rehabilitation period, two of the participants regained the ability to modulate some of the leg movements during EES, indicating that residual natural pathways were present and that their recovery might be boosted with biomimetic EES. Indeed, the same group had demonstrated previously that spatiotemporal neuromodulation therapies engaging muscle synergies improve motor control after SCI.^9,10^ To enhance the recovery further and enable the patients with SCI to regain natural movement, a combination of biological repair interventions such as hPSC-based cellular therapy and neurorehabilitation supported by EES are probably the currently most promising way forward.

## Revolution of iPSC-based Therapy—From a Personalized to the “Off-the-Shelf” Approach

Following the discovery of iPSCs,^11^ the initial dream of personalized therapy was quickly shattered when developers faced the manufacturing costs. Only 8 years after the iPSCs were discovered, the world’s first iPSC-based clinical trial was initiated in Japan for the treatment of age-related macular degeneration of the retina.^12^ The patient had to wait over 10 months from the skin biopsy till the surgery. Reprogramming, differentiation, and Quality Control/Quality Assurance took their toll. The costs of the autologous transplantation of iPSC-derived retinal pigment epithelium (RPE) cells amounted to approximately USD 1 million.^13^ Obviously, this was not sustainable.

To reduce the costs of an allogeneic approach, the ideal donors would be healthy with homozygous human leukocyte antigen (HLA)-A, HLA-B, and HLA-DR. It is estimated that 10, 75, and 140 cell lines would match approximately 50%, 80%, and 90% of the Japanese population.^14-16^ Donor recruitment was achieved through the collaboration with the Japan Red Cross, Japan Marrow Donor Program, and several Japanese cord blood banks because they already had HLA typing data available for all stored blood samples. In a relatively short period, 36 donors agreed to participate in the project; 20 of them were homozygous for all 6, and 15 donors were homozygous for the 5 HLA loci.^13^

Clinical grade iPSC lines with three distinct homozygous HLA haplotypes, matching approximately 32% of the Japanese population, were released in 2015. In March 2017, one of these lines was used in the first allogeneic transplantation,^17^ which was mimicking the procedure of the previous trial. The surgery time was shortened to about 1 month, and the overall cost was under USD 200 000 per patient.^13^

Although this strategy might work for a highly homogeneous population such as the Japanese, high ethnic diversity in other countries, such as in Europe or US, makes this task nearly insurmountable. The only plausible alternative would be to create hPSC lines with the capacity to evade the immune system—so-called, universal donor hPSC lines.

## Chasing a Holy Grail—Universal Donor hPSC

A central role in allogeneic rejection is played by HLA class I molecules through their presentation of peptide antigens to CD8^+^ T cells. To be expressed on the cell surface, they all require β_2_-microglobulin (B2M), which is coded by a non-polymorphic gene. Several groups have generated B2M^−/−^ hPSCs, eliminating class I surface expression and preventing the stimulation of allogeneic CD8^+^ T cells, including University of Washington, Seattle, spin-off *Universal Cells*^18^ and *Advanced Cell Technology*.^19^

This approach, however, did not work. HLA class I-negative cells were lysed by natural killer (NK) cells through the missing self-response. University of Washington/*Universal Cells* team solved the problem.^20^ Using adeno-associated virus (AAV), they re-engineered B2M^−/−^ hPSCs to express HLA-E as a single-chain protein fused to B2M, and thereby created the cells that express minimally polymorphic HLA-E as their only surface HLA class I molecule.

According to the *Universal Cells* website, the company is also working on a strategy of inactivating HLA class II molecules DP, DQ, and DR, which present peptides to CD4^+^ T cells. They are composed of polymorphic alpha and beta chains and do not use B2M for cell surface expression. The common feature of class II molecules is that their promoters require the same set of transcription factors (*RFX5*, *RFXANK*, *RFXAP*, or *CIITA*). Mutations in these factors would prevent the expression of HLA class II molecules.

*Astellas Pharma* has acquired both companies; in February 2016, *Advanced Cell Technology*, which was renamed *Ocata Therapeutics*, and 2 years later, in February 2018, *Universal Cells*. By the end of 2021, *Astellas* has been sponsoring 8 clinical trials with hPSC, although all of them are evaluating hESC-based therapy (Table 1).

Although the strategy seemed to be well designed, it had some drawbacks. The HLA-E is the canonical activator of KLRC2 (NKG2C), a dominant activating receptor found on human NK cells. NK cells preferentially express several calcium-dependent (C-type) lectins, which have been implicated in the regulation of NK cell function. The cells engineered to over-express HLA-E, while effective in inhibiting KLRC1+ (NKG2A+) NK cells, were unable to inhibit but instead activated KLRC2+ (NKG2C+) NK cells.^21^ These data suggested that other strategies are warranted.

It has been suggested that overexpression of NK inhibitory molecules in hPSC might allow the cells to “hide” from allogeneic T-cell recognition while inhibiting their NK-mediated lysis. Indeed, mouse iPSCs lose their immunogenicity when major histocompatibility complex (MHC) class I and II genes are inactivated and NK inhibitory ligand CD47 is over-expressed.^22^ However, the data from the human system did not match expectations. The expression of the main CD47 interactor signal regulatory protein alpha (SIRPA) is mostly restricted to macrophages and dendritic cells and not human NK cells, and the observed effects of this immune-modulating strategy in the mouse system could offer only partial or incomplete immune evasion in the human system.^23^ Furthermore, the entire strategy of overexpression of NK inhibitory molecules has a caveat. The expression patterns of NK inhibitory receptors are heterogenous,^24^ and each NK inhibitory receptor is not expressed on all NK cells. Therefore, it is not easy to suppress NK cell activation in its entirety.^25^

*Fate Therapeutics* (CA, US; https://fatetherapeutics.com), known for its transgene-free reprogramming technology yielding ground state-like pluripotency stem cells,^26^ went a step ahead of its competitors. Their iPSC-derived NK (iNK) cell therapy is multiplexed with a novel combination of immune-evasion modalities: (i) B2M knockout to prevent CD8^+^ T-cell-mediated rejection; (ii) class II transactivator (CIITA) knockout to prevent CD4^+^ T-cell-mediated rejection; and (iii) CD38 knockout to enable combination therapy with anti-CD38 monoclonal antibodies, which can be administered to deplete host alloreactive lymphocytes, including both NK and T cells.^23,27^ When given in a combination with checkpoint inhibition therapies, such as PD-L1/PD-1 blockade, iNK cells further enhanced inflammatory cytokine production and exerted stronger cytotoxicity against an array of hematologic and solid tumors.^28^ The company is currently a direct sponsor of 9 and a partner in additional 4 clinical trials involving their iNK cells (Table 1).

## A Paradigm Shift?

The standard strategy for a cutting-edge cancer treatment requires extracting T cells from a patient, engineering them ex vivo, in a laboratory, to produce chimeric antigen receptors (CARs) on the surface that will enable them to latch on cancer cells, and then reintroducing them back to the patient. The entire process is expensive, which makes the therapy itself difficult to afford. A single dose of Kymriah (tisagenlecleucel) for patients in pediatric care is priced at USD 475 000 and Yescarta (axicabtagene ciloleucel) for certain types of non-Hodgkin lymphoma at USD 373 000.^29^ These prices rival some of the most expensive medical procedures such as a kidney transplant that is priced at USD 415 000. Due to the shorter time and lower costs of manufacturing, universal donor hPSC-derived immune therapy of cancer is likely to replace such personalized CAR T-cell therapy in future. There is no need to extract T cells and engineer them ex vivo. The off-the-shelf iNK cells could be available and ready to use right away. Any point of care that can perform a blood transfusion would be able to administer the iNK therapy too.

A new technology that can bypass ex vivo part, nanomedicine-mediated in vivo reprogramming, has recently emerged: a therapeutic approach to generate transient CAR T cells in vivo by delivering modified messenger RNA (mRNA) in T-cell-targeted lipid nanoparticles (LNPs) for the treatment of cardiac fibrosis has been reported.^30^ This is only a preclinical study in a mouse model, and we cannot assume that it will work safely in humans. If the technology ends up being safe and effective enough in the treatment of human diseases, it may reduce the importance of the universal donor hPSC-derived immune therapy. However, due to its transient nature, this approach would not be applicable for regenerative therapies of solid organs.

## How About hPSC-based Therapy of Diabetes?

Hundreds of articles have been published on stem cell-based treatment of diabetes (PubMed search with key words “stem cell therapy diabetes” yielded more than 5000 articles). However, despite all these predictions, the stem cell-based therapy of diabetes is still in clinical trials and out of reach. Insulin, a hundred years following its discovery, and islet transplantation that started about 20 years ago, are still the only effective treatment of diabetes. The encapsulation device as a strategy of delivering cellular therapy for diabetes was pioneered more than a decade ago. New Zealand-based *Living Cell Technologies* (https://lctglobal.com) successfully demonstrated the effectiveness of alginate-encapsulated neonatal porcine pancreatic islets in the first approved xeno-therapy trial. However, the improvement was only short-lived, and this approach was not pursued. The development of a combined advanced therapy medicinal products (ATMP), especially encapsulation devices, for the therapy of diabetes is clearly warranted.

It seems that *ViaCyte* (CA, US; https://viacyte.com), a pioneer in the development of hPSC-based therapy of diabetes, has been the most successful. They changed the design of their proprietary encapsulation devices several times; the most recent one, composed of a medical-grade plastic called expanded polytetrafluoroethylene (ePTFE), was developed in collaboration with *Gore* (DE, US; www.gore.com). *ViaCyte* has recently reported interim results of a landmark stem cell therapy trial for type I diabetes.^31,32^ The insulin-secreting cells were delivered to the patients in macroencapsulation device. The results from the first cohort of a phase I/II trial showed that the treated patients were on their way of achieving insulin independence. The implants were safe, and the data demonstrated evidence of meal-regulated insulin secretion by differentiated stem cells in patients.

In February 2022, *ViaCyte* (CA, US; https://viacyte.com) and *CRISPR Therapeutics* (Switzerland; www.crisprtx.com) announced a phase I clinical trials of VCTX210, an hESC-based therapy for type 1 diabetes without the need for immunosuppression. The CyT49 hESC line lacks the *B2M* gene and expresses a transgene encoding CD274 also known as programmed death ligand 1 (PD-L1) to further protect from T-cell attack. Thus, gene-edited, immune-evasive, hPSC-based cellular therapy is not reserved only for the treatment of malignancies.^33,34^

## The Future of hPSC-based Therapies

It is quite likely that the upward trend will continue and that a number of hPSC-based clinical trials will grow rapidly in the next few years. US, Japan, and China will remain the leading countries. The closest “competitor,” the UK, is still lagging behind. The primary reasons for segregation of the three leading counties are the costs of development and manufacturing of the hPSC-based therapies in line with the safety standards required by the regulatory agencies. Only well-financed businesses in countries with a developed infrastructure and large capital investments available can take advantage in the burgeoning field.

Inevitably, genetically engineered universal donor hPSCs and combined ATMPs will dominate the future of hPSC-based therapy. New quality standards can be established only by bringing together the most recent technology and diverse scientific state-of-the-art expertise in biotechnology, biomaterial sciences, and artificial intelligence. Working together across disciplines will foster the development and implementation of existing and new technologies, thus speeding up progress toward the use of hPSC-based therapies in translational medicine.

## Data Availability

No new data were generated or analyzed in support of this research.

## References

[1] Ilic D , Devito L, Miere C, et al. Human embryonic and induced pluripotent stem cells in clinical trials. Br Med Bull. 2015;116:19-27. 10.1093/bmb/ldv045 26582538

[2] Kobold S , Guhr A, Mah N, et al. A manually curated database on clinical studies involving cell products derived from human pluripotent stem cells. Stem Cell Rep. 2020;15:546-555. 10.1016/j.stemcr.2020.06.014 PMC7419703 32679065

[3] Newsroom Shepherd Center. Shepherd center patient treated in Geron clinical trial. October 11, 2010. Accessed March 10, 2022. https://news.shepherd.org/shepherd-center-patient-treated-in-geron-clinical-trial/

[4] Lebkowski J. GRNOPC1: the world’s first embryonic stem cell-derived therapy. Interview with Jane Lebkowski. Regen Med. 2011;6(Suppl 6):11-13. 10.2217/rme.11.77 21999256

[5] Kaiser J. Embryonic stem cells. Researchers mull impact of Geron’s sudden exit from field. Science. 2011;334:1043. 10.1126/science.334.6059.1043 22116849

[6] United States Securities and Exchange Commission, Asterias Biotherapeutics, Inc. 2017. 8-K Current report. EXHIBIT 99.2. Accessed March 10, 2022. https://sec.report/Document/0001140361-17-037043/ex99_2.htm

[7] Sugai K , Sumida M, Shofuda T, et al. First-in-human clinical trial of transplantation of iPSC-derived NS/PCs in subacute complete spinal cord injury: study protocol. Regen Ther. 2021;18:321-333. 10.1016/j.reth.2021.08.005 34522725 PMC8427225

[8] Rowald A , Komi S, Demesmaeker R, et al. Activity-dependent spinal cord neuromodulation rapidly restores trunk and leg motor functions after complete paralysis. Nat Med. 2022;28:260-271. 10.1038/s41591-021-01663-5 35132264

[9] Wagner FB , Mignardot JB, Le Goff-Mignardot CG, et al. Targeted neurotechnology restores walking in humans with spinal cord injury. Nature. 2018;563:65-71. 10.1038/s41586-018-0649-2 30382197

[10] Courtine G , Sofroniew MV. Spinal cord repair: advances in biology and technology. Nat Med. 2019;25:898-908. 10.1038/s41591-019-0475-6 31160817

[11] Takahashi K , Yamanaka S. Induction of pluripotent stem cells from mouse embryonic and adult fibroblast cultures by defined factors. Cell. 2006;126:663-676. 10.1016/j.cell.2006.07.024 16904174

[12] Mandai M , Watanabe A, Kurimoto Y, et al. Autologous induced stem-cell-derived retinal cells for macular degeneration. N Engl J Med. 2017;376:1038-1046. 10.1056/NEJMoa1608368 28296613

[13] Umekage M , Sato Y, Takasu N. Overview: an iPS cell stock at CiRA. Inflamm Regen. 2019;39:17. 10.1186/s41232-019-0106-0 31497180 PMC6717959

[14] Nakatsuji N , Nakajima F, Tokunaga K. HLA-haplotype banking and iPS cells. Nat Biotechnol. 2008;26:739-740. 10.1038/nbt0708-739 18612291

[15] Saito MK , Matsunaga A, Takasu N et al. Donor recruitment and eligibility criteria for HLA-homozygous iPS cell bank in Japan. In: Ilic D, ed. Stem Cell Banking. Springer; 2014:67-76. 10.1007/978-1-4939-0585-0_7

[16] HLA Laboratory. Haplotype Frequency. Accessed March 10, 2022. http://hla.or.jp/med/frequency_search/en/haplo/

[17] Sugita S , Mandai M, Hirami Y, et al. HLA-matched allogeneic iPS cells-derived RPE transplantation for macular degeneration. J Clin Med. 2020;9:2217. 10.3390/jcm9072217 PMC7408794 32668747

[18] Riolobos L , Hirata RK, Turtle CJ, et al. HLA engineering of human pluripotent stem cells. Mol Ther. 2013;21:1232-1241. 10.1038/mt.2013.59 23629003 PMC3677304

[19] Feng Q , Shabrani N, Thon JN, et al. Scalable generation of universal platelets from human induced pluripotent stem cells. Stem Cell Rep. 2014;3:817-831. 10.1016/j.stemcr.2014.09.010 PMC4235139 25418726

[20] Gornalusse GG , Hirata RK, et al. HLA-E-expressing pluripotent stem cells escape allogeneic responses and lysis by NK cells. Nat Biotechnol. 2017;35:765-772. 10.1038/nbt.3860 28504668 PMC5548598

[21] Williams AM , Hayama K, Pan Y, et al. A novel stealth strategy that activates adoptively transferred allogeneic immune cells and avoids rejection for off-the-shelf cell-based cancer therapy. Blood. 2021;138(Suppl 1):4800. 10.1182/blood-2021-153614

[22] Deuse T , Hu X, Agbor-Enoh S, et al. The SIRPα-CD47 immune checkpoint in NK cells. J Exp Med. 2021;218:e20200839. 10.1084/jem.20200839 33416832 PMC7802363

[23] Mbofung RM , Williams AM, Hayama K, et al. Off-the-shelf, iPSC-derived CAR-NK cells multiplexed-engineered for the avoidance of allogeneic host immune cell rejection. Blood. 2021;138(Suppl 1):4800. 10.1182/blood-2021-153484

[24] Horowitz A , Strauss-Albee DM, Leipold M, et al. Genetic and environmental determinants of human NK cell diversity revealed by mass cytometry. Sci Transl Med. 2013;5:208ra145. 10.1126/scitranslmed.3006702 PMC3918221 24154599

[25] Koga K , Wang B, Kaneko S. Current status and future perspectives of HLA-edited induced pluripotent stem cells. Inflamm Regen 2020;40:23. 10.1186/s41232-020-00132-9 33014207 PMC7528263

[26] Valamehr B , Robinson M, Abujarour R, et al. Platform for induction and maintenance of transgene-free hiPSCs resembling ground state pluripotent stem cells. Stem Cell Rep. 2014;2:366-381. 10.1016/j.stemcr.2014.01.014 PMC3964282 24672758

[27] Woan KV , Kim H, Bjordahl R, et al. Harnessing features of adaptive NK cells to generate iPSC-derived NK cells for enhanced immunotherapy. Cell Stem Cell. 2021;28:2062-2075. 10.1016/j.stem.2021.08.013 34525347 PMC8642276

[28] Cichocki F , Bjordahl R, Gaidarova S, et al. iPSC-derived NK cells maintain high cytotoxicity and enhance in vivo tumor control in concert with T cells and anti-PD-1 therapy. Sci Transl Med. 2020;12:eaaz5618. 10.1126/scitranslmed.aaz5618 33148626 PMC8861807

[29] Mukherjee S. The promise and price of cellular therapies. The New Yorker. 2019. Published in the print edition of the July 22, 2019, issue (p. 48-57), with the headline “New Blood”. Accessed March 10, 2022. https://www.newyorker.com/magazine/2019/07/22/the-promise-and-price-of-cellular-therapies

[30] Rurik JG , Tombacz I, Yadegari A, et al. CAR T cells produced in vivo to treat cardiac injury. Science. 2022;375:91-96. 10.1126/science.abm0594 34990237 PMC9983611

[31] Shapiro AMJ , Thomson D, Donner TW, et al. Insulin expression and C-peptide in type 1 diabetes subjects implanted with stem cell-derived pancreatic endoderm cells in an encapsulation device. Cell Rep Med. 2021;2:100466. 10.1016/j.xcrm.2021.100466 35028608 PMC8714853

[32] Ramzy A , Thompson DM, Ward-Hartstonge KA, et al. Implanted pluripotent stem-cell-derived pancreatic endoderm cells secrete glucose-responsive C-peptide in patients with type 1 diabetes. Cell Stem Cell. 2021;28:2047-2061. 10.1016/j.stem.2021.10.003 34861146

[33] Viacyte. CRISPR Therapeutics and ViaCyte present positive in vitro data towards a potential immune-evasive cell replacement therapy for diabetes at EASD 2019. September 17, 2019. Accessed May 6, 2022. https://viacyte.com/press-releases/crispr-therapeutics-and-viacyte-present-positive-in-vitro-data-towards-a-potential-immune-evasive-cell-replacement-therapy-for-diabetes-at-easd-2019/

[34] Viacyte. CRISPR Therapeutics and ViaCyte, Inc. announce first patient dosed in phase 1 clinical trial of novel gene-edited cell replacement therapy for treatment of type 1 diabetes (T1D). February 2, 2022. Accessed May 6, 2022. https://viacyte.com/press-releases/crispr-therapeutics-and-viacyte-inc-announce-first-patient-dosed-in-phase-1-clinical-trial-of-novel-gene-edited-cell-replacement-therapy-for-treatment-of-type-1-diabetes-t1d/

